# Colorectal cancer screening: Understanding the health literacy needs of hispanic rural residents

**DOI:** 10.3934/publichealth.2019.2.107

**Published:** 2019-04-01

**Authors:** Angelina R. Wittich, L. Aubree Shay, Belinda Flores, Elisabeth M. De La Rosa, Taylor Mackay, Melissa A. Valerio

**Affiliations:** 1 UTHealth School of Public Health in San Antonio, Health Promotions and Behavioral Science, San Antonio, TX., USA; 2 South Coastal AHEC (Area Health Education Center), University of Texas Health Science Center at San Antonio, Corpus Christi, TX., USA; 3 Institute for Integration of Medicine & Science-Community Engagement, University of Texas Health Science Center at San Antonio, San Antonio, TX., USA

**Keywords:** colorectal cancer, screening, Hispanics, rural, health literacy

## Abstract

**Purpose:**

Hispanics residing in rural areas are among those who are *least likely* to be screened for colorectal cancer (CRC) and more likely to present with late stage CRC than other racial or ethnic groups. We conducted a pilot study utilizing a mixed-method approach to explore perceptions of CRC and CRC screening among Hispanic adults residing in South Texas rural communities and to identify health literacy needs associated with CRC screening uptake.

**Methods:**

A convenience sample of 58 participants, aged 35–65, were recruited to complete questionnaires and participate in focus groups, ranging in size from 4 to 13 participants. Six focus groups were conducted across 3 adjacent rural counties. A semi-structured moderator's guide was designed to elicit discussion about participants' experiences, knowledge, and perceptions of CRC and CRC screening.

**Findings:**

Lack of knowledge of CRC and CRC screening as cancer prevention was a common theme across focus groups. A majority, 59%, reported never been screened. Thirty-nine percent reported they had been screened for colon cancer and 5% reported they did not know if they had been screened. Participants with lower educational levels perceived themselves at high risk for developing CRC polyps, would not want to know if they had CRC, and if they did have CRC, would not want to know until the very end. Limited information about CRC and CRC screening, a lack of specialized providers, limited transportation assistance, and compromised personal privacy in small-town medical facilities were perceived to be barriers to CRC screening.

**Conclusions:**

Low screening rates persist among rural Hispanics. Improving CRC screening literacy and addressing factors unique to rural Hispanics may be a beneficial strategy for reducing screening disparities in this at-risk population.

## 1. Introduction

The leading cause of death among Hispanics in the United States is cancer [1]. An estimated one out of every three Hispanic men and women will be diagnosed with cancer over their life course [1]. In the United States, colorectal cancer (CRC) is the second leading cause of all cancer deaths [2], and the third leading cause of cancer deaths among Hispanic men and women, respectively [1]. According to the Texas Cancer Registry, Hispanics have lower CRC mortality rates than Whites or African Americans. However, in the Public Health Region 11 (PH 11) where the study participants reside, Hispanics make up 85% of the population and have higher incidence and mortality rates than Whites, which make up 13% of the population. Although African Americans generally have higher incidence and mortality rates than Hispanics and Whites, in Public Health Region 11, they represent only 1% of the population. While some might conclude that Hispanics have lower mortality rates due to a younger aged population, according to the U.S. Census Bureau, median age is similar for Texas and the three counties under study (i.e., 34 versus 34, 28, and 35 years of age, respectively). In Texas, CRC is the second leading cause of deaths, second to lung cancer [2].

Colorectal Cancer screening is critical for early detection of cancerous lesions and increasing survivorship [3]. Inasmuch as Hispanics are least likely to be screened for CRC [4],[5], it is important to identify the underlying factors that lead to low screening rates among this large and growing segment of the population for several reasons. First, cancer is the leading cause of death for Hispanics, whereas heart disease is the leading cause of death among Whites [2]. Second, compared to Whites, Hispanics are more likely to be diagnosed at the late-stage of cancer [1], making them more vulnerable to mortality. Third, there is a disparity in mortality trends. According to a 2017 American Cancer Society report, mortality is decreasing at a slower pace among Hispanics compared to Whites and Blacks [2]. Lower screening rates and late-stage diagnosis contributes to this mortality disparity.

In spite of concerted efforts to promote CRC screening across the United States [6], Hispanics lag behind other racial and ethnic groups in being up-to-date with United States Preventive Services Task Force (USPSTF) screening recommendations [4],[7]–[10]. The USPSTF recommends CRC screening in adults, beginning at age 50 years and continuing until age 75 years, using fecal occult blood testing, sigmoidoscopy, or colonoscopy. Compared to Whites, Blacks and Asians, CRC screening rates increased the least for Hispanics [4],[11]. In fact, between 2003 and 2013, for adults aged 50–75, screening rates increased from 41% to 60% for Whites, 35% to 58% for Blacks, 27% to 50% for Asians, but the rates for Hispanics only increased from 27% to 41% [12]. In 2016, among individuals aged 50–75 years residing in the metropolitan statistical area where our participants were recruited, only 69% reported ever having a colonoscopy [13]. There are also CRC screening disparities between urban and rural populations, with rural residing Hispanics having the lowest prevalence of CRC screening [5],[4],[14]. For rural Whites and Blacks, screening prevalence was 39% and 35%, respectively, and 28% for Hispanics [15]. These rates are lower than urban residing populations whereby screening prevalence among Whites, Blacks, and Hispanics are 45%, 42%, and 30%, respectively[14] Whereas multi-component interventions that are culturally tailored have been deemed important strategies for addressing disparities in CRC screening [15],[16], they have had little to modest effect on screening behaviors among Hispanics [17]–[19].

Given that Hispanics are less likely to get screened [4] and more likely to present with late stage CRC [2],[20], identifying underlying factors associated with screening behavior is crucial for developing effective strategies and interventions to increase screening rates, improve survivability, and thereby impact health disparities. The present study focused on exploring experiences, knowledge, and perceptions of CRC and CRC screening and health literacy deficits that might contribute to low screening rates among Hispanics residing in rural communities of the South Texas Coastal Bend.

## 2. Methods

### 2.1. Participant Recruitment

Participants were recruited in collaboration with the South Coastal Area Health Education Center (SC-AHEC), a community partner of the UTHealth School of Public Health in San Antonio, located in Nueces County. All study recruitment materials were drafted and shared with the SC-AHEC for their review. Recommendations from the staff of SC-AHEC for tailoring the recruitment and discussion guides to the local population and making them more culturally-relevant were adopted. Recruitment flyers were distributed by SC-AHEC staff at local churches and community centers. A convenience sample was comprised of individuals responding to the flyers and screened by the SC-AHEC staff for study inclusion. Inclusion criteria included adults aged 35–65 residing in Jim Wells, Nueces, and Kleberg counties of the South Texas Coastal Region. Study protocol and materials were approved by the Institutional Review Board's Committee for the Protection of Human Subjects of the University of Texas Health Science Center at Houston (HSC-SPH-14-0283). All SC-AHEC staff involved in the study participated in human subject protection training.

### 2.2. Procedures and Data Collection

A total of six discussion groups, ranging in size from four to thirteen participants (fifty-eight total participants), in five communities across the three adjacent rural counties were held. Upon completing a consent form, focus group participants were asked to complete a questionnaire containing items on the participants' demographics, medical history, job and work environment, perceptions and attitudes about CRC and screening. To assess perceived risks of developing CRC and perceptions of screening efficacy we used survey questions developed and validated by Vernon and colleagues [21]. Participant attitudes regarding CRC were assessed with survey questions developed by McCaffery and colleagues [22].

Focus group sessions began after participants completed demographic and behavior questionnaires. The principal investigator (MAV) and a SC-AHEC (BF) representative conducted each focus group session using a semi-structured moderator's guide designed to elicit discussion among the participants about their experiences with and perceptions of cancer in general, and CRC, specifically. Questions included: “When you hear the words colon cancer, what words, feelings or ideas come to mind?”, “Are some groups or types of people more likely to get colorectal cancer?”, “When it comes to not getting colorectal cancer are there certain things that people can do to not get it?”, “What are your thoughts on finding colorectal cancer earlier versus finding it later?”, and “Are there things that make it difficult for people to go get the exam”? The focus groups were conducted in a manner to respectfully honor participants' thoughts and comments and ensure their anonymity. All study participants were English speaking. Each focus group was conducted in English, lasted approximately an hour and a half, and was audio recorded. Study participants received a small meal prior to the focus group session and a $20 gift card to a local store as an incentive for their participation.

### 2.3. Analysis

Statistical Package for the Social Sciences (SPSS) was used to manage and analyze the data from the fifty-eight questionnaires. After each data entry session, a 25% random spot check was conducted to check for accuracy. Frequencies and means were calculated to describe demographic characteristics, perceptions about CRC screening, and CRC attitudes. Fisher's exact test and independent sample t-tests were used to test for differences in perceptions and attitudes by level of education among categorical variables and continuous variables, respectively.

Each of the six discussion group sessions were transcribed verbatim. The audio recordings and corresponding transcriptions were reviewed by two graduate assistants for accuracy. A grounded theory approach and an iterative coding process was utilized to analyze the contents of each of the transcripts [23]. Each transcript was initially reviewed and indexed to identify statements reflecting recurrent categories, concepts, or themes regarding aspects of cancer risk perceptions, knowledge of and barriers to CRC screening, rural residency that were common across the groups. The transcripts were then independently coded by two graduate assistants and a staff medical sociologist. All coding was reviewed and discrepancies were resolved by the research team. The principal investigator supervised the coding process to finalize codes, concepts, and themes and reviewed disparities in coding for final agreement [24].

## 3. Results

### 3.1. Participant characteristics and survey results

A majority of the participants were Hispanic (96.6%), female (79.3%), and had a high school education or less (64.9%) (Table 1). The mean age was fifty-five years and 41.1% reported an income of less than $20,000.

**Table 1.** Socio-demographic characteristics and screening behavior (N = 58)*.

Characteristic	% (n)
Ethnicity	
Non-Hispanic	3.4 (2)
Hispanic	96.6 (56)
Sex	
Male	20.7 (12)
Female	79.3 (46)
Education	
Less than high school	29.8 (17)
GED/high school graduate	35.1 (20)
Post high school technical school	1.8 (1)
Some college	19.3 (11)
College degree or higher	14.0 (8)
Household Income	
Less than $20,000	41.1 (23)
$20,000–$39,999	23.2 (13)
$40,000–$59,999	14.2 (8)
$60,000 or More	21.5 (12)
Age (mean, range)	55.4 (35–88)
Ever screened for colorectal cancer	
Yes	39.3 (22)
No	55.4 (31)
Don't Know	5.4 (3)

* Missing data: Education n = 1, Household Income n = 2, Ever Screened n = 2.

Slightly more than one-third (39%) reported they had been screened for colon cancer, 55% reported never been screened and 5% reported they did not know if they had been screened. Only seven participants (12.1%) reported a family history of colon cancer. Compared to those having more education (i.e., more than high school education), a higher percentage of participants with less education (i.e., high school education or less) reported believing that they were at high risk for developing CRC polyps (48% vs 20%), and had a preference for remaining uninformed about a cancer diagnosis (Likert score of 2.9 vs 1.9), and would not want to know about a CRC diagnosis until the very end (Likert score of 2.4 vs 1.7) (Table 2). Responses did not vary greatly by age, sex, or income level.

**Table 2.** Colorectal cancer screening perceptions and cancer attitudes (N=58).

Perceived Susceptibility	High school education or less %True (n)	More than high school education % True (n)
I believe that the chance I might develop colorectal cancer is high.	25.0 (9)	14.3 (3)
I think that it is very likely that I will develop colorectal cancer or polyps.	48.5 (16)	23.8 (5)
I believe that the chance I will develop colorectal cancer polyps is high.	48.6 (17)	20.0 (4)

Perceived Screening Efficacy	High school education or less% True (n)	More than high school education% True (n)

I believe that if I had a normal cancer screening test, I wouldn't have to worry about developing colorectal cancer.	57.1 (20)	38.1 (8)
I think that when colorectal polyps are found and removed, colorectal cancer can be prevented.	80.6 (29)	76.2 (16)
I believe that when colorectal cancer is found early, it can be cured.	97.2 (35)	90.5 (19)

Cancer Attitudes*	High school education or lessmean (sd)	More than high school educationmean (sd)

When it comes to cancer, If I had something wrong, I would rather know as soon as possible.	4.6 (0.8)	4.7 (0.9)
I am afraid to think about colorectal cancer.	3.7 (1.4)	3.0 (1.4)
If I had cancer, I would rather not know about it.	2.9 (1.5)	1.9 (1.2)
If I had colorectal cancer, I would not want to know until the very end.	2.4 (1.4)	1.7 (0.7)
The thought of colorectal cancer scares me.	3.8 (1.3)	3.5 (1.2)

* Cancer attitude items were scored on a 5-point Likert Scale ranging from Strongly Disagree (1) to Strongly Agree (5).

### 3.2. Qualitative Results

The following section highlights four themes emerging from the qualitative analysis.

#### 3.2.1. Knowledge of CRC is limited

Overall participants demonstrated a lack of knowledge about CRC or an understanding of the importance of CRC screening to detect CRC and improve survival.

“I would think it's a family history. Down the line if your mother had it, I would say go get checked and the same with heart, or with diabetes, or you know . . . it's a pattern that follows.”

Interestingly, many participants accurately identified risk factors for CRC including family history of CRC, diet, risky health behaviors, ethnic or racial background, and gender were accurately identified as risk factors.

“Yes . . . that depends on like the race, or what they eat … too much fat … too much grease and things like that . . . too much beer . . . smoking cigarettes.”

“Is it mostly known for men to get it? … Cause men drink more beer.”

Participants perceived environmental conditions as risk factors for CRC. While environmental conditions may indeed contribute to illness and certain cancers, these perceptions illustrate a lack of understanding of CRC.

“I've lived in areas that are called colonias, and you can't help but call them cancer clusters because the ladies that have lived next door to each other, both of them suffer from breast cancer . . . They dump fertilizer into the soil . . . They've lived there all their lives, and you see this, you know, immense amounts of cancer and strokes . . . ”

“. . . when it would rain like this, when we were little we used to go play in the temples. Now you don't even see none of that because . . . everything makes you sick right . . . everything is dirty. . . You have to wear gloves to walk around . . .”

#### 3.2.2. Cancer screening is beneficial

There was a widespread recognition among participants that cancer screening is beneficial. Having a family history of CRC was perceived to be a motivating factor for undergoing CRC screening. Although most participants expressed an awareness of CRC screening as beneficial for diagnosing, treatment, and improving survivability, a lack of understanding of the procedure as a cancer detecting process was also evident. For example, as one participant noted:

“I know you have to go and get a colonoscopy but I always thought of that as just getting checked for . . . making sure your intestines are doing fine…everything is running smooth the way it's supposed to . . .”

#### 3.2.3. Screening barriers are difficult to overcome

Participants discussed both individual- and system-level factors as barriers to CRC screening. Common personal barriers included a difficult preparation process, embarrassment and discomfort with the screening procedure, and a lack of caregiver support to deal with the emotional and physical stress of undergoing a colonoscopy, especially for those living in remote rural areas. A unique barrier to residents of small communities with few local health care facilities is the lack of privacy. The quote below represents concerns expressed by study participants across focus groups.

“Well I know here in the community because of the hospital and it being so small, everybody knows everybody, and it's like, “Oh no, I don't want them checking my behind…I know this person works in the ER . . . because it's a very private matter.”

Participants identified typical system level factors as barriers, including a lack of insurance, high deductible rates, lack of transportation and accessibility to specialty physicians. However, participants singled out transportation and accessibility as significant barriers unique to those living in rural communities.

“Cause you living in south Texas you got a lot of little ranches where those people . . . out there that cannot make it into town. . . And there's, believe it or not there's a lot of people that need help out there. You know they're not fortunate enough to be living in town or being able to come in town every day like we do.”

“. . . we don't have anything here in Kingsville, like you have to go out of town and a lot of these people don't have transportation either, so it's kinda hard. You know and then it's like oh we have to drive them to (another town). . .”

#### 3.2.4. CRC screening can be facilitated

Participants voiced the need for, and importance of, organized efforts by healthcare and government systems to promote and facilitate CRC screenings across communities. Lowering the cost of screening for low income and underinsured populations was viewed as a practical way to facilitate screenings.

Participants cited media (i.e., public service announcements) as an influential factor in drawing attention to the benefits of screening for breast cancer and prostate cancer. However, with respect to CRC, participants reported a lack of media campaigns advocating CRC screening. Across focus groups, participants expressed a desire to learn more about CRC and CRC screening. Presenting information at local community-based health fairs was viewed as an ideal way to reach community members given that these types of events are well received in rural areas. The importance of using *easy to understand* bilingual formats to inform the community and promote CRC screening was also expressed across groups. Another example of how the media could be utilized to maximize advocacy in a cultural context is reflected in the quote below.

“I think too that with more education . . . maybe more information on the Spanish stations . . . I know my mom was big into the novellas, so it has to be with education maybe at one of the senior centers or something.”

## 3. Discussion

The purpose of this study was to explore perceptions of CRC and CRC screening among Hispanic adults residing in South Texas rural communities and to identify health literacy needs associated with CRC screening uptake. Texas is among those states having the lowest percentage of individuals aged 50–75 who are up-to-date with CRC screening [26]. Whereas Hispanics make up 39% of the population in Texas, in the southernmost region of Texas, where 84% of the population is Hispanic [26], CRC screening rates are among the lowest in the state, with less than half ever receiving CRC screening [13]. Whites and African Americans represent 42% and 13%, respectively, of the Texas population. In general, Hispanics have lower mortality rates compared to Whites and Blacks [1],[2]. Reported death rates for CRC for men are 17% (White), 26% (Black), and 15% (Hispanic) [2]. Colorectal cancer death rates for women are 12% (White), 17% (Black), and 9% (Hispanic) [2]. In spite of lower mortality rates among Hispanics, improved understanding of barriers to CRC screening among the largest ethnic group in Texas, not only addresses an important public health concern for a significant portion of the population in Texas, it contributes to the literature on cancer screening disparities.

Limited health literacy has been associated with low rates of preventive cancer screenings [27]–[30] and is a common barrier to CRC screening for Hispanics [27],[31]. Our finding that over 60% of study sample had either never been screened for colon cancer or did not know if they had been screened are consistent with previously reported data on Texas Hispanics [32]. Similarly, our results are reflective of previous research demonstrating an association between low educational attainment and lower cancer screening rates among Hispanics and Latinos [7],[33],[34]. For example, in the study population most participants (65%) had high school education or less and only 39% reported ever being screened. Our finding that those with less education remain uninformed about a CRC diagnosis and screening suggests that much work is needed to improve health literacy of CRC and screening among rural Hispanic populations. A health literacy need among this population in that may be a lack of understanding that screening significantly reduces mortality rates [35]. Moreover, findings such as a preference to remain uninformed about a CRC diagnosis “until the end” suggests a need for improved strategies for communicating the value of CRC screening in culturally tailored messages among rural residing Hispanics. Given that tailored messaging has been shown to be an effective means for increasing CRC screening [36]–[38], even among those at greater risk for CRC, such as individuals whose family members have CRC [39], integrating messages that have relevancy and salience for Hispanics into a multi-component intervention is important because Hispanics are often at greater risk for late stage diagnosis [2],[20].

Regarding perceived benefits of screening, we found that study participants do have an appreciation for the value of cancer screening in preventing and curing cancer, largely as a result of media advocacy. Participants identified the media's influential role in their decisions to get screened for breast and prostate cancer. However, participants reported that much less attention is given to CRC screening across the media spectrum. Further research is needed to examine the role and impact of media advocacy on CRC screening rates for rural residing individuals.

Our findings that transportation issues and accessibility to providers for rural residing individuals is consistent with the literature [40],[41]. The fact that gastroenterologists were available in only one of the five cities in our study, speaks to the difficulty of obtaining CRC screening and reducing CRC mortality for rural residents [42]. A recent study demonstrated that death from CRC was reduced the most if a colonoscopy was performed by a gastroenterologist. According to the study authors, individuals having a colonoscopy performed by a gastroenterologist had a lower risk of dying from CRC than if the colonoscopies were performed by primary-care physicians or surgeons [43].

While having health insurance eliminates a barrier for obtaining CRC screening [12], having a low income presents a barrier for many individuals, including Hispanic individuals [27]. The low income status of our study participants is problematic in that obtaining CRC screening involves not only the cost of the screening, but also the potential loss of wages for time off to have the procedure, and particularly for rural residing individuals, transportation expenses to a distant urban facility that provides screening. Although our results reveal that income was not significantly associated with obtaining CRC screening, participants did cite cost of the screening as a substantial barrier.

Limitations. This small, pilot study was not powered to conduct statistical analyses. Additionally, we used a convenience sample that may not generalize to those who chose not to participate. Future studies should confirm our findings in a larger, random sample. An additional potential limitation of the study is that all of the participants were English speaking and we cannot generalize our findings to those rural Hispanics who do not speak English. While we understand that non-English speaking individuals may be more vulnerable given a potential language barrier, English is spoken by a majority of residents in the study area [44]. Another limitation is that measures and reports of CRC screening were self-reported and not corroborated by medical records. However, self-reports of CRC screening have been found to be a valid and reliable source[45]–[47] and our findings of low CRC screening rates among the Hispanic study population is consistent with previous research [4],[7],[9]. In addition, although we did not assess health literacy of the participants, low screening rates have been associated with inadequate health literacy [27],[29],[31],[49],[50], limited education attainment [32],[52],[53], a lack of knowledge of CRC [29],[50] and rural residency [54].

## 4. Conclusion

To eliminate the CRC screening disparities gap between Hispanics, Whites and Blacks, we must identify and better understand barriers [7],[54]–[58] at the local community level. A recent study demonstrated that frequent exposure to CRC screening information results in significantly greater screening participation. Sources of screening information included news reports, advertisements sponsored by the American Cancer Society, the *Screen for Life* campaign sponsored by the Centers for Disease Control and Prevention (CDC), and patient education materials [59]. The results of this study demonstrate that frequent exposure to screening via mass media increases the likelihood of obtaining CRC screening. Given that our study participants expressed a desire for exposure to various formats of CRC screening campaigns, a coordinated, multicomponent effort with local media outlets and community partners may be a strategic way to address the health literacy needs associated with improved utilization of CRC screening in rural Hispanic communities, particularly among those living in non-metropolitan areas who are at most risk for not being screened [5],[60].
